# SLC14A1 and TGF-β signaling: a feedback loop driving EMT and colorectal cancer metachronous liver metastasis

**DOI:** 10.1186/s13046-024-03114-8

**Published:** 2024-07-27

**Authors:** Yixun Zhang, Yumeng Yang, Xuan Qi, Peng Cui, Yi Kang, Haiyi Liu, Zhigang Wei, Haibo Wang

**Affiliations:** 1 Department of Colorectal Surgery, Cancer Hospital Affiliated to Shanxi Medical University, Shanxi Province Cancer Hospital, Shanxi Hospital Affiliated to Cancer Hospital, Chinese Academy of Medical Sciences, Taiyuan, China; 2 Department of Biochemistry and Molecular Biology, School of Basic Medical Sciences, Capital Medical University, No.10 Xitoutiao, You An Men Wai, Beijing, 100069 China; 3 Laboratory for Clinical Medicine, Capital Medical University, No.10 Xitoutiao, You An Men Wai, Beijing, China; 4 Department of General Surgery, State Key Lab of Digestive Health, National Clinical Research Center for Digestive Diseases, Beijing Friendship Hospital, Capital Medical University, Beijing, China; 5 Department of Gastroenterology, Cancer Hospital Affiliated to Shanxi Medical University, Shanxi Province Cancer Hospital, Shanxi Hospital Affiliated to Cancer Hospital, Chinese Academy of Medical Sciences, Taiyuan, China; 6 Hepatobiliary and Pancreatic Surgery and Liver Transplantation Center, First Hospital of Shanxi Medical University, 85 Jiefang Nan Lu, Taiyuan, 030001 Shanxi China; 7 Beijing Laboratory of Oral Health, Capital Medical University, No.10 Xitoutiao, You An Men Wai, Beijing, China

**Keywords:** SLC14A1, TGF-β/Smad signaling, Colorectal cancer, Metachronous liver metastasis

## Abstract

**Background:**

Colorectal cancer (CRC) metachronous liver metastasis is a significant clinical challenge, largely attributable to the late detection and the intricate molecular mechanisms that remain poorly understood. This study aims to elucidate the role of Solute Carrier Family 14 Member 1 (SLC14A1) in the pathogenesis and progression of CRC metachronous liver metastasis.

**Methods:**

We conducted a comprehensive analysis of CRC patient data from The Cancer Genome Atlas and GSE40967 databases, focusing on the differential expression of genes associated with non-metachronous liver metastasis and metachronous liver metastasis. Functional assays, both in vitro and in vivo, were performed to assess the biological impact of SLC14A1 modulation in CRC cells. Gene set enrichment analysis, molecular assays and immunohistochemical analyses on clinical specimens were employed to unravel the underlying mechanisms through which SLC14A1 exerts its effects.

**Results:**

SLC14A1 was identified as a differentially expressed gene, with its overexpression significantly correlating with poor relapse-free and overall survival. Mechanistically, elevated SLC14A1 levels enhanced CRC cell invasiveness and migratory abilities, corroborated by upregulated TGF-β/Smad signaling and Epithelial-Mesenchymal Transition. SLC14A1 interacted with TβRII and stabilized TβRII protein, impeding its Smurf1-mediated K48-linked ubiquitination and degradation, amplifying TGF-β/Smad signaling. Furthermore, TGF-β1 reciprocally elevated SLC14A1 mRNA expression, with Snail identified as a transcriptional regulator, binding downstream of SLC14A1’s transcription start site, establishing a positive feedback loop. Clinically, SLC14A1, phosphorylated Smad2, and Snail were markedly upregulated in CRC patients with metachronous liver metastasis, underscoring their potential as prognostic markers.

**Conclusions:**

Our findings unveil SLC14A1 as a critical regulator in CRC metachronous liver metastasis, providing novel insights into the molecular crosstalk between SLC14A1 and TGF-β/Smad signaling. These discoveries not only enhance our understanding of CRC metachronous liver metastasis pathogenesis, but also highlight SLC14A1 as a promising target for therapeutic intervention and predictive marker.

**Supplementary Information:**

The online version contains supplementary material available at 10.1186/s13046-024-03114-8.

## Introduction

Colorectal cancer (CRC) remains one of the most prevalent and lethal malignancies worldwide [1]. A significant challenge in the management of CRC is the occurrence of liver metastasis, which substantially impact patient prognosis and survival [2]. Among these, metachronous liver metastasis, which emerge within a timeframe of six months to two years after the resection of primary tumor, pose a unique clinical challenge. Despite advancements in surgical techniques and systemic chemotherapy, the prognosis for patients with metachronous liver metastasis remains guarded, primarily due to the late detection and the intricate nature of tumor dissemination [3].

Currently, the molecular mechanisms driving the pathogenesis of metachronous liver metastasis are not fully elucidated, creating a significant barrier to the development of effective therapeutic strategies and reliable predictive markers [4, 5]. This gap in understanding underscores the necessity for intensive research focused on the molecular and cellular pathways involved in the metastatic spread to the liver following primary CRC treatment. Such advancements would not only facilitate early intervention but also pave the way for the development of targeted therapies, ultimately improving the survival and quality of life of CRC patients.

Several genetic and epigenetic alterations have been implicated in CRC liver metastasis, including mutations in key tumor suppressor genes and oncogenes, aberrant signaling pathways, and modifications in the tumor microenvironment. These complex molecular interactions modulate cancer cell proliferation, evasion of apoptosis, angiogenesis, invasion, and metastasis [6]. Among them, the role of the Transforming Growth Factor-Beta (TGF-β) signaling pathway and its orchestration of the Epithelial-Mesenchymal Transition (EMT) has been brought to the forefront of cancer research [7–11]. In the early stages of CRC, the TGF-β pathway exerts tumor-suppressive effects, characterized by cell cycle arrest and the induction of apoptosis. However, as the disease progresses, cancer cells often develop resistance mechanisms that mitigate the suppressive impact of TGF-β, transforming this pathway into a facilitator of tumor progression and metastasis. A pivotal mechanism through which the TGF-β pathway promotes CRC metastasis is the induction of EMT [11]. EMT is a biological process in which epithelial cells undergo a phenotypic switch, adopting mesenchymal attributes that confer enhanced migratory, invasive, and anti-apoptotic properties. This transition is pivotal in embryonic development, tissue repair, and particularly, in the context of CRC, tumor invasion and metastasis [9]. EMT is characterized by the downregulation of epithelial markers such as E-cadherin and the upregulation of mesenchymal markers including N-cadherin and vimentin. These alterations facilitate the dissociation of cancer cells from the primary tumor mass, promoting their invasion into surrounding tissues and subsequent metastasis to distant organs, including the liver [12].

Evidence linking the TGF-β pathway to EMT induction and CRC metastasis is growing [13–15]. TGF-β signaling is known to stimulate the expression of transcription factors including Snail, Slug, and Twist, pivotal regulators of the EMT process [16]. This relationship underscores the complex interplay between TGF-β signaling and EMT in facilitating the invasive and metastatic potential of CRC cells. It is thus imperative to delineate these molecular interactions meticulously, as they present potential targets for therapeutic intervention aimed at curtailing CRC progression and metastasis.

In our pursuit to delineate the molecular intricacies associated with CRC metachronous liver metastasis, we identified Solute Carrier Family 14 Member 1 (SLC14A1) as a differentially expressed genes (DEGs), potentially serving as a crucial modulator in this complex process. SLC14A1, a urea transporter protein, has hitherto been primarily associated with renal functions and bladder cancer [17–19]. However, SLC14A1’s role in cancers, particularly CRC, is yet enigmatic, necessitating comprehensive investigations to unravel its functional and mechanistic implications. In this study, we embark on a meticulous exploration, employing a combination of bioinformatics, cellular, molecular, and clinical approaches to elucidate the role and mechanistic underpinnings of SLC14A1 in promoting CRC metachronous liver metastasis. We aim to offer invaluable insights, bridging existing knowledge gaps, and providing a foundation for the development of targeted therapeutic and diagnostic strategies to mitigate the incidence and improve the prognosis of CRC metachronous liver metastasis. Our findings could potentially unveil novel molecular targets, opening avenues for personalized medicine applications, and enhancing the survival and quality of life for CRC patients enduring the ordeal of liver metastasis.

## Materials and methods

### Antibodies and reagents

The antibodies used in this study were sourced from the following providers: anti-SLC14A1 antibody (Abcam, ab103295/ab238721), anti-Snail antibody (Abcam, ab85936), anti-Ubiquitin antibody (Abcam, ab134953), anti-Smurf1 antibody (Abcam, ab300408), anti-TGFβ Receptor II antibody (Abcam, ab259360), anti-HA antibody (Abcam, ab9110), anti-Smad2 antibody (CST, #5339), anti-Smad3 antibody (CST, #9523), anti-pSmad2 antibody (CST, #8828), anti-pSmad3 antibody (CST, #9520), anti-E-Cadherin antibody (CST, #3195), anti-N-Cadherin antibody (CST, #13,116), anti-Vimentin antibody (CST, #5741), anti-β-Tubulin antibody (CST, #2146), anti-Lamin B1 antibody (CST, #13,435), anti-Flag antibody (Sigma-Aldrich, St. Louis, MO) and anti-His antibody (MBL, Nagoya, Japan), anti-β-actin antibody (Bioss, Beijing, China). Additionally, HRP-conjugated secondary antibodies were obtained from ZSGB-BIO (Beijing, China).

In addition, the study utilized the following reagents: MG132 (HY-13259, MedChemExpress, Shanghai, China); TGF-β1 (HY-P7117, MedChemExpress, Shanghai, China); SB431542 (HY-10431, MedChemExpress, Shanghai, China), anti-TGF-β1 Recombinant Antibody (HY-P99305, MedChemExpress, Shanghai, China), Cycloheximide (GLPBIO, Montclair, CA); protein A/G-agarose (Santa Cruz Biotechnology) and Matrigel (Corning, New York).

### Bioinformatics analysis

For this study, RNA-seq data and clinical information of CRC were obtained from The Cancer Genome Atlas (TCGA), accessed via the cBioPortal (http://www.cbioportal.org/). Additionally, CRC microarray data were sourced from the Gene Expression Omnibus (GEO), specifically dataset GSE40967 (https://www.ncbi.nlm.nih.gov/geo/geo2r/?acc=GSE40967). To identify differentially expressed genes (DEGs) in CRC primary tumor, contrasting non-metachronous liver metastasis with metachronous liver metastasis cases, we employed the NetworkAnalyst and GEO2R analyzer online tools. DEGs were selected based on stringent criteria, including an adjusted P-value of less than 0.05 and an absolute Log2 (fold change) greater than 0.2. For visualization of the overlap between datasets, Venn diagrams were generated using the Venny website (https://bioinfogp.cnb.csic.es/tools/venny/). Patients were stratified into the metachronous liver metastasis group based on the following criteria: (a) no liver metastasis at initial diagnosis; (b) underwent curative surgery for CRC with a pathological M stage of M0; (c) developed liver metastasis six months post-surgery; (d) had not received neoadjuvant chemotherapy, neoadjuvant chemoradiotherapy, or other preoperative treatments.

Gene Set Enrichment Analysis (GSEA) was conducted to evaluate the statistical significance of enrichment in gene sets from the Molecular Signatures Database (http://www.gsea-msigdb.org/). We employed the default parameters for GSEA, including 1000 permutations. Enrichment results were considered statistically significant if the false discovery rate was below 0.25 and the P-value was less than 0.05.

### Cell culture

Human CRC cell lines, including SW480, HCT116, HT29, RKO, LoVo, and the kidney cell line 293 T, were acquired from the National Infrastructure of Cell Line Resource, Beijing, China. The cell lines SW480, HCT116, and RKO were cultured in RPMI-1640 medium, whereas HT29, LoVo, and 293 T cells were maintained in DMEM. Both media were supplemented with 1% penicillin/streptomycin and 10% fetal bovine serum, ensuring optimal growth conditions. The cell culture environment was consistently maintained at 37 °C in a humidified atmosphere containing 5% CO_2_.

Prior to their use in experiments, the cell lines underwent authentication using short tandem repeat (STR) DNA fingerprinting. This process was essential to confirm the identity and purity of the cell lines. Additionally, all cell lines were tested and found negative for mycoplasma contamination, ensuring the validity of experimental results. To maintain genetic stability and reduce the risk of phenotypic drift, the passage number of all cell lines was strictly limited to a maximum of 30 continuous passages.

### Generation of stable cell lines and transfection

The overexpression construct for SLC14A1 or Snail were engineered using lentiviral vectors supplied by Gene-Chem, located in Shanghai, China. SLC14A1 or Snail knockdown was achieved via shRNA-mediated lentiviral vectors, also sourced from Gene-Chem. Stable cell lines were then developed to express either SLC14A1 or Snail cDNA or shRNA specific to SLC14A1 or Snail. The infection was followed by a selection phase using 2 μg/mL puromycin, obtained from Solarbio, Beijing, China, which lasted for a duration of 7 days. Verification of SLC14A1 or Snail overexpression or knockdown efficiency was conducted through immunoblotting assays. HA-Ub and HA-Ub/K48R were purchased from UNIBIO company (www.unibio.net). Transient transfection was performed using the Lipofectamine 3000 reagent, with the procedure carried out according to the instructions provided in the Lipofectamine 3000 transfection manual.

### Matrigel transwell invasion assay

The invasion assay was conducted in accordance with the protocol provided by Costar (Cambridge, USA). Modified Boyden chambers featuring 8-μm pore filter inserts designed for 24-well plates (Costar) were utilized. These filters were initially coated with 100 μl of ice-cold 10% Matrigel diluted in medium. Subsequently, 1 × 10^5^ cells suspended in 200 μl of serum-free medium were seeded into the upper chamber, while the lower chamber was filled with 600 μl of complete medium. The chambers were then incubated at 37 °C in an atmosphere containing 5% CO_2_ for 20 h. Post-incubation, cells were fixed using 4% paraformaldehyde for 10 min. Cells remaining on the upper surface of the filter were gently removed using cotton swabs. The cells that had successfully invaded through the filter were stained with a 0.5% crystal violet solution for 10 min. Stained cells were imaged using a Nikon compound microscope, capturing four representative fields per membrane. The number of cells in each field was quantified. This experiment was independently replicated three times for statistical robustness.

### Wound-healing migration assay

Cell seeding was performed in 6-well plates, with a density of 4 × 10^5^ cells per well, and incubated for 12 h to attain 90–100% confluence. Subsequently, cells were conditioned in RPMI-1640 medium, both supplemented with 1% FBS, for an additional 12 h. Wound creation was achieved by gently scratching the cell monolayer with a 200 μl pipette tip. Following this, cells were rinsed twice with phosphate-buffered saline (PBS) and then continued to be cultured in their media supplemented with 1% FBS at 37 °C. The wound area was imaged at 12-h intervals. This assay was independently conducted three times for each cell line to ensure repeatability. Quantification of cell migration was performed by measuring the wound area at various time points and comparing these measurements to the initial wound width.

### RNA extraction and quantitative real-time PCR (qPCR) analysis

Total RNA was isolated from cell samples using the RAN-Quick Purification Kit (Yishan Biotechnology, #RN001, Shanghai, China), strictly following the manufacturer's protocol. The synthesis of first-strand cDNA was performed using HiScript® III RT SuperMix for qPCR (+ gDNA wiper) (Vazyme, #R323, Nanjing, China). Quantitative PCR was executed as per the guidelines provided by Life Technologies. Gene expression analysis was conducted in triplicate employing FastStart Universal SYBR Green Master with ROX (#04913850001, Roche Applied Science) on an Applied Biosystems 7900HT Fast Real-Time PCR System. The relative expression levels of the genes were quantified using the 2 − ΔΔCt method. β-actin was employed as an endogenous control for normalization purposes. The specific primers used for qPCR are detailed in Additional file 1: Table S1.

### Animal experimental procedures

The animal study protocols were granted approval by the Institutional Animal Care and Use Committee. Male or female athymic BALB/c nude mice, aged 4–6 weeks, were selected for the experiments. These mice were housed under specific pathogen-free conditions at a controlled room temperature.

In the intra-splenic injection procedure, the mice were anesthetized using pentobarbital sodium (1.5 mg/20 g) via intraperitoneal injection. A small incision was made in the left abdominal flank to expose the spleen. For the experiment, 3 × 10^6^ SW480 cells, either with or without SLC14A1 overexpression, were suspended in 50 µl of HBSS. This cell suspension was then slowly injected into the spleen using a 32 G needle. Post-injection, the spleen was gently compressed with a cotton swab for 5 min to prevent extravasation and ensure the migration of tumor cells to the liver. After a period of 4 weeks, the mice were euthanized, and liver metastasis in both groups were counted and subjected to statistical analysis.

The orthotopic caecal injection model of CRC liver metastasis in mice was established as follows. BALB/c nude mice were anesthetized and immobilized on the surgical table. The surgical area was prepared by disinfection. A midline abdominal incision was made to access the peritoneal cavity and locate the caecum. The caecum was exteriorized and stabilized on a surgical platform. CRC cells (HCT116-shSLC14A1, HCT116-shCtrl) suspended in sterile PBS were injected into the caecal wall at a predetermined site using an insulin syringe. A volume of 20 µl (1 × 10^6^ cells) was injected into the caecal wall and the caecum was returned to the peritoneal cavity, and the peritoneum and skin were closed with sutures. Postoperatively, mice were monitored for recovery and general health. After a period of 60 days, the mice were euthanized and their liver weight and body weight were recorded. In addition, fix the liver with 4% paraformaldehyde and perform HE staining. Finally, perform statistical analysis on the obtained data.

### Enzyme-linked Immunosorbent Assay (ELISA)

TGF-β1 concentration in cell culture supernatant was measured using an ELISA kit according to the manufacturer's instructions (Beyotime, PT880, Beijing). CRC cells were treated by corresponding agents for 24 h then cultured in FBS-free medium for 72 h. Then, the culture medium was collected and centrifuged at 1500 rpm for 5 min. TGF-β1 in the supernatant was detected.

### Nuclear‑cytoplasmic separation

Nuclear-cytoplasmic separation was conducted on two transfected cell lines (SW480 and HCT116) following a standardized protocol. The cells were collected and treated with cytoplasmic protein extraction reagent A containing phenylmethanesulfonyl fluoride (PMSF), followed by addition of cytoplasmic protein extraction reagent B and a 10-min incubation on ice. Subsequently, the samples were centrifuged at 12,000 g and 4 °C for 5 min to separate cytoplasmic proteins in the supernatant from nuclear proteins in the pellets. Nuclear protein extraction was carried out by adding a nuclear protein extraction reagent to the pellets and incubating on ice for 30 min with intermittent shaking. After centrifugation at 1000 g and 4 °C for 10 min, the supernatants containing nuclear proteins were collected. The concentrations of cytoplasmic and nuclear proteins were determined using a BCA assay and further analyzed by Western blotting.

### Western blot analysis

Cellular lysates were prepared using RIPA buffer supplemented with both protease inhibitor cocktail and phosphatase inhibitor cocktail. These lysates were then utilized for Western blot analysis. The procedure commenced with denaturation of the lysates by boiling at 95 °C for 5 min, followed by their resolution via sodium dodecyl sulfate–polyacrylamide gel electrophoresis (SDS-PAGE). Subsequently, the separated proteins were transferred onto PVDF membranes. These membranes were blocked for 1 h at room temperature using 5% non-fat milk dissolved in TBS buffer containing 0.1% Tween-20 (TBST). After blocking, the membranes were cut based on protein molecular weights and incubated overnight at 4 °C with specific primary antibodies. Following this incubation, membranes were washed with TBST and then incubated with horseradish peroxidase (HRP)-conjugated secondary antibodies, room temperature for 1 hour. Immunoreactive bands were detected and quantified using NIH Image 1.62 software (National Institutes of Health, Bethesda, MD).

### Coimmunoprecipitation (Co-IP)

Co-IP was conducted following a previously established protocol [20]. The procedure initiated with cell lysis, for which cells were lysed in a suitable lysis buffer. Post-lysis, the cell lysate was subjected to clarification to remove cell debris. The immunoprecipitation reactions were set up using specific antibodies tailored to the target proteins and the clarified cell lysates. These mixtures were then incubated to allow for the formation of antibody-protein complexes. Subsequent to this incubation, protein A/G-agarose beads were added to each reaction mixture. The mixtures were then incubated with gentle rotation to facilitate binding. Following the incubation, the complexes bound to the agarose beads were isolated by centrifugation. These immunoprecipitants were thoroughly washed with PBS to remove non-specifically bound proteins. The final step involved resolving the bound proteins by SDS-PAGE, followed by immunoblot analysis to detect and analyze the target proteins.

### Luciferase reporter assay for Snail regulation of SLC14A1 mRNA

The assessment of Snail's regulatory effects on SLC14A1 mRNA was conducted using a dual-luciferase reporter assay. For this purpose, the promoter region of SLC14A1, extending from 2000 base pairs (bp) upstream to 500 bp downstream of the transcription start site (TSS), along with its various mutants, were cloned into the pGL3-Basic firefly luciferase reporter plasmid. These constructs were then co-transfected into 293 T cells, either with Snail or a control vector. Post-transfection, both firefly and Renilla luciferase activities were quantified using the dual-luciferase reporter assay system provided by Promega (USA). The relative luciferase activity was determined by normalizing the firefly luciferase activity against the Renilla luciferase activity. This normalization helps to control for transfection efficiency and cell viability, providing a more accurate measure of the regulatory effects under investigation.

### Collection of CRC surgical specimens

The study involved a retrospective collection of surgical specimens and associated clinical data from patients diagnosed with CRC. These specimens and data were sourced from patients who received treatment at the Cancer Hospital Affiliated to Shanxi Medical University during the period from 2013 to 2018. Inclusion criteria for the study were as follows: 1) Patients who underwent curative resection of the primary CRC lesion, with postoperative pathology confirming CRC; 2) Regular follow-up examinations within five years post-surgery to determine the presence or absence of metachronous liver metastasis; 3) Complete clinical records and postoperative follow-up information. Exclusion criteria: 1) Presence of synchronous liver metastasis; 2) Patients who received neoadjuvant chemotherapy, neoadjuvant chemoradiotherapy, or other preoperative treatments.

In total, the study encompassed 230 patients, consisting of 128 males and 102 females, with an average age of 59.98 ± 9.31 years. The research protocol and the implementation plan adhered strictly to the ethical guidelines outlined in the Declaration of Helsinki. The Ethics Committee of the Cancer Hospital Affiliated to Shanxi Medical University granted formal approval for this study (2022JC22). Furthermore, written informed consent was meticulously obtained from all participants, authorizing the use of their surgical specimens and the publication of associated data in this research.

### Immunohistochemistry (IHC) staining

IHC assay was described previously [21]. In brief, tissue sections were initially incubated with specific primary antibodies overnight at 4 °C within a humidified chamber. This step was followed by a 15-min incubation with horseradish peroxidase (HRP)-conjugated secondary antibodies. For signal detection, a DAB (diaminobenzidine) detection kit (ZSGB-BIO) was employed, and counterstaining was conducted using haematoxylin (Solarbio). After dehydration, the slides were sealed with resin and scanned using an Aperio GT450 digital slide scanner. Image acquisition was carried out using Image Scope software. The evaluation of SLC14A1, p-SMAD2 and Snail staining in the tissue sections was independently conducted by two experienced pathologists who were blinded to the clinical and pathological data of the patients. Staining intensity was graded on a scale from 0 to 3: 0 denoting no staining, 1 indicating weak staining, 2 representing moderate staining, and 3 for strong staining. A semi-quantitative assessment was also performed to evaluate the proportion of positive tumor cells, using a scoring system ranging from 0 to 4, where 0 indicates no positive tumor cells, 1 for 1–25% positive cells, 2 for 26–50%, 3 for 51–75%, and 4 for over 75% positive cells. For a comprehensive quantification of SLC14A1, p-SMAD2, and Snail expression, an H-score was calculated for each sample. This score was derived by multiplying the intensity score by the percentage score.

### Statistical analysis

All statistical analyses were conducted utilizing GraphPad Prism software. For continuous variables, the analysis of variance (ANOVA) or t-tests were employed, depending on the data distribution. In cases where data did not follow a Gaussian distribution, appropriate nonparametric tests were applied. For assessing the correlation between variables, Pearson's correlation coefficient was used for data with a Gaussian distribution, whereas Spearman's correlation coefficient was utilized for non-Gaussian distributed data. Survival rates across different groups were compared using the log-rank test, based on Kaplan–Meier survival curves. The results are presented as mean ± standard error of the mean (SEM). For all tests, a P-value of less than 0.05 (two-tailed) was set as the threshold for statistical significance.

## Results

### Identification of SLC14A1 as a key modulator in CRC metachronous liver metastasis

**Fig. 1:**
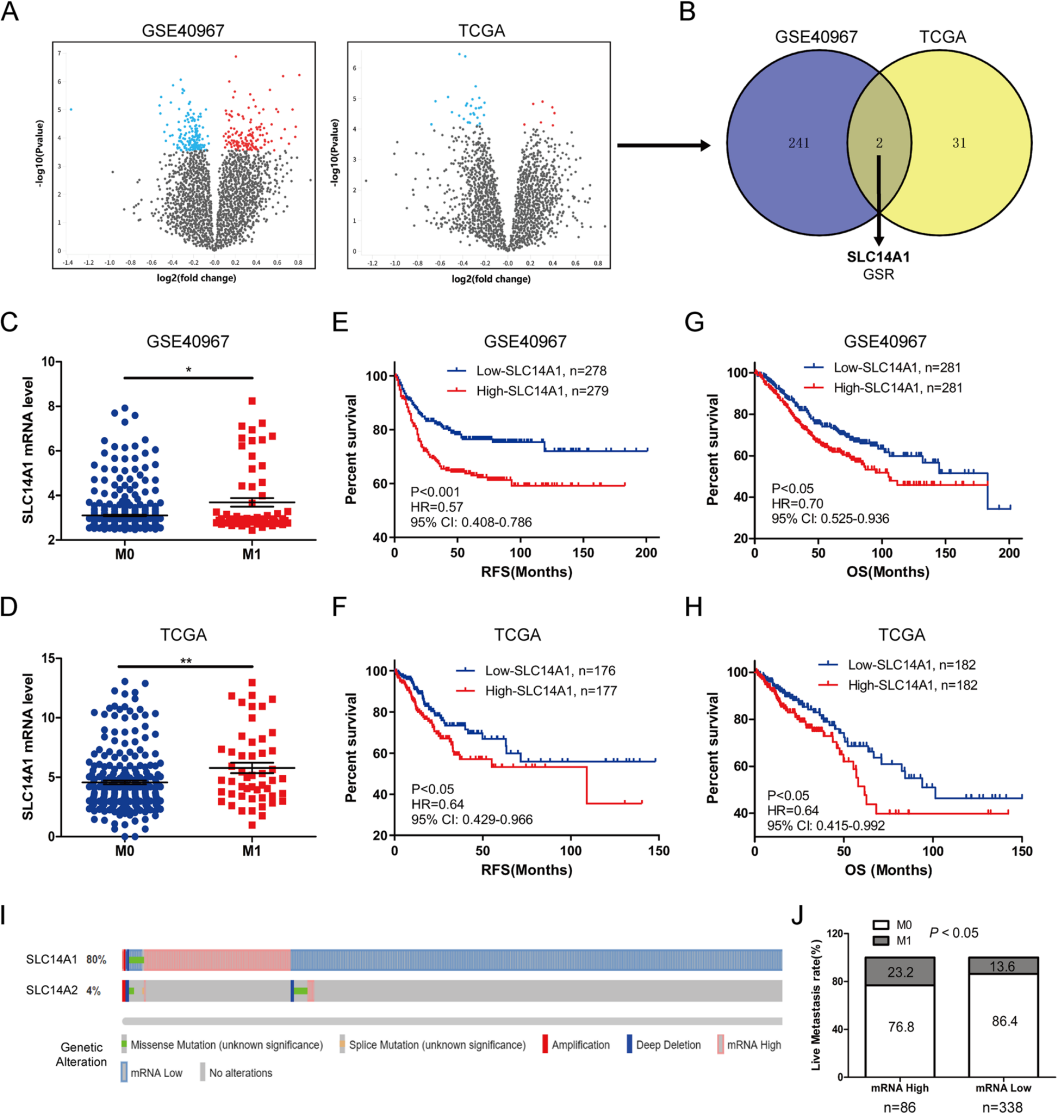
Elevated SLC14A1 Expression in CRC Tissues Is Linked to Metachronous Liver Metastasis and Reduced Survival Rates. **A** Differentially expressed genes (DEGs) between CRC samples with and without metachronous liver metastasis from GSE40967 and TCGA datasets is depicted in a volcano plot. The x-axis represents the log2 fold change, and the y-axis represents the -log10 P value. **B** A Venn diagram highlights two common DEGs, including SLC14A1 and Glutathione Reductase (GSR), identified in both GSE40967 and TCGA datasets. **C**, **D** Dot plots illustrate elevated SLC14A1 mRNA levels in CRC tissues with liver metastasis (M1) compared to those without metastasis (M0), based on GSE40967 and TCGA data. Data are mean ± SEM, analyzed by a 2-tailed unpaired t-test (*, *P* < 0.05, **, *P* < 0.01). **E–H** Kaplan–Meier (KM) survival curves demonstrate the impact of SLC14A1 mRNA expression on recurrence-free survival (RFS) and overall survival (OS) in CRC patients from GSE40967 and TCGA, analyzed by log-rank test. CI: Confidence interval, HR: Hazard Ratio. **I** The genetic alteration frequencies of SLC14A1 and SLC14A2 in CRC patients are presented using TCGA database information. SLC14A1 mRNA expression is defined by 'mRNA expression z-scores relative to normal samples (log RNA Seq V2 RSEM)'. The high and low expression groups for SLC14A1 mRNA were stratified based on z-scores (± 2.0). **J** CRC patients exhibiting high SLC14A1 mRNA expression levels are associated with a higher incidence of liver metastasis, as determined by chi-square test

Patients were categorized into groups with and without metachronous liver metastasis for differentially expressed genes (DEGs) analysis conducted on two independent databases, GSE40967 and TCGA. This analysis resulted in the identification of 243 and 33 DEGs, respectively (Fig. 1A). An intersection of the DEGs from both databases highlighted SLC14A1 and GSR (glutathione-disulfide reductase) as common DEGs (Fig. 1B). Further analysis of both databases indicated a significant upregulation of SLC14A1 mRNA levels in the liver metastasis group (Fig. 1C, D). Kaplan–Meier survival analysis demonstrated that patients with higher SLC14A1 expression had significantly poorer relapse-free survival (RFS) (Fig. 1E, F) and overall survival (OS) (Fig. 1G, H) compared to those with lower expression.

In addition, we investigated genetic alteration in the SLC14A family, including SLC14A1 and SLC14A2, amongst CRC patients in the TCGA database, utilizing the cBioPortal database. The findings revealed an 80% genetic alteration rate of SLC14A1 in CRC tissues, manifested primarily as upregulation and downregulation of mRNA, while the genetic alteration rate for SLC14A2 stood at a mere 4% (Fig. 1I). Notably, the liver metastasis rate was 23.2% in patients with upregulated SLC14A1 and 13.6% in those with downregulated expression. Chi-square test analysis validated the statistical significance of the disparity in metastasis rates between the two groups, where the high SLC14A1 group showed a higher rate of liver metastasis (Fig. 1J).

### SLC14A1 promotes colorectal cancer liver metastasis (CRCLM) in vitro and in vivo

**Fig. 2:**
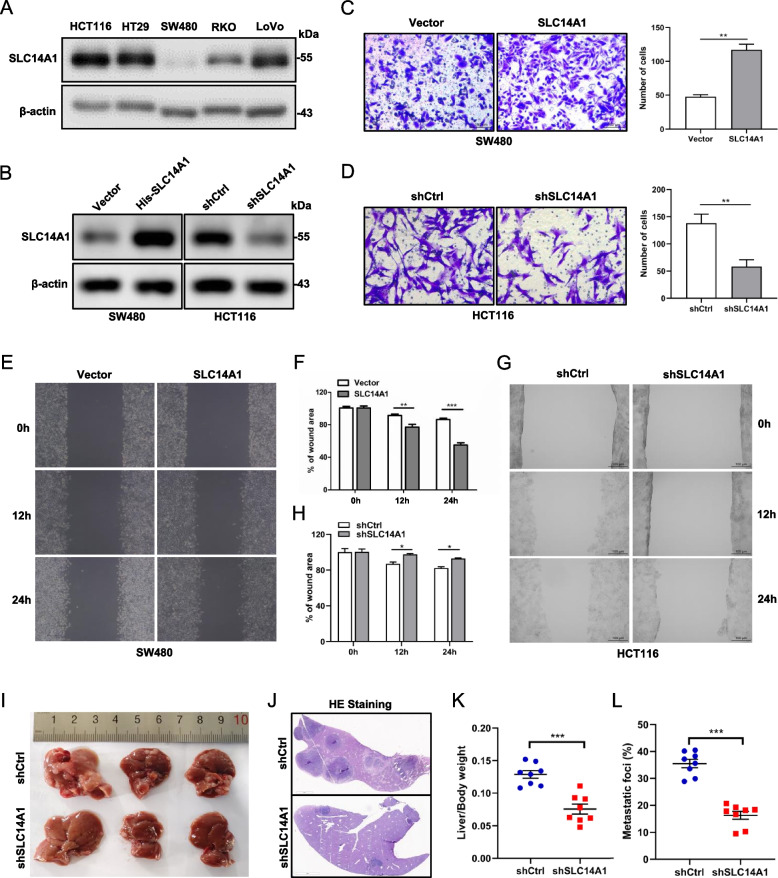
SLC14A1 Enhances Invasion, Migration, and Metastatic Potential in CRC Cells. **A** Western blot analysis was used to evaluate SLC14A1 protein levels across various CRC cell lines. **B** Stable overexpression and knockdown of SLC14A1 in SW480 and HCT116 cells were confirmed via western blotting. **C**,** D** Manipulation of SLC14A1 expression altered CRC cells' invasive capabilities. Transwell assays were utilized to evaluate invasion capacity, with representative images displayed on the left (scale bars: 100 μm). The quantified number of invading cells is shown on the right (*P* < 0.01). **E–H** SLC14A1 overexpression or knockdown significantly influenced the migratory behavior of CRC cells. **I-L** Knockdown SLC14A1 inhibited liver metastasis in cecal orthotopic injection model of liver metastasis using the HCT116 cell line. BALB/C mice (*n* = 8, per group) were injected with either HCT116/shCtrl or HCT116/shSLC14A1 cells. Representative pictures (**I**) and H&E section (**J**) harvested from BALB/C mice (shCtrl vs. shSLC14A1), and liver/body weight (**K**) and liver CRC foci coverage (**L**) on 60 days after HCT116 injection. Data are presented as mean ± SEM, *p* < 0.001***

To delineate the functional role of SLC14A1 in CRCLM, we embarked on a comprehensive series of experimental analyses. Initially, the protein expression of SLC14A1 across various CRC cell lines was ascertained through Western blot, revealing discernible variations in expression levels (Fig. 2A). To facilitate a robust assessment of functional implications, SW480 cells characterized by low endogenous SLC14A1 expression were genetically modified to overexpress SLC14A1. Conversely, SLC14A1 was knocked down in HCT116 cells, where its expression is intrinsically elevated (Fig. 2B). These manipulations culminated in the establishment of stable cell lines that offered a contrasting spectrum of SLC14A1 expression for subsequent analyses.

In the realm of cellular invasiveness, insights from transwell assays were illuminating. SW480 cells with SLC14A1 overexpression exhibited a marked augmentation in invasive potential (Fig. 2C), whilst the invasive capability of HCT116 was notably attenuated upon SLC14A1 knockdown (Fig. 2D). These observations were mirrored in scratch wound healing assays, where enhanced SLC14A1 expression expedited wound closure in SW480 cells, indicative of increased migratory capacity (Fig. 2E, F). In contrast, the migratory prowess of HCT116 cells was compromised following SLC14A1 reduction (Fig. 2G, H). To further corroborate our findings through in vivo experiments, we established a surgically orthotopic implantation model of CRCLM in mice by cecal injection of HCT116 cells stably knocked down for SLC14A1 or control. The results demonstrated that knockdown of SLC14A1 significantly reduced the liver metastatic capability of HCT116 cells compared to the control group (Fig. 2I, J). Mice with SLC14A1 knockdown exhibited an increased ratio of liver weight to total body weight and a higher ratio of metastatic lesion area to total liver area (Fig. 2K, L). Additionally, we constructed a splenic injection model of CRCLM using SW480 cells overexpressing SLC14A1 or control. The results showed that mice in the SLC14A1 overexpression group had a significantly increased number of liver metastatic lesions (Additional file 2: Fig. S1).

These evidences from both in vitro and in vivo studies compellingly attest to the pivotal role of upregulated SLC14A1 in augmenting the invasiveness and migratory capacity of CRC cells.

### SLC14A1 augments EMT through TGF-β/Smad2/3 signaling in CRCLM

**Fig. 3:**
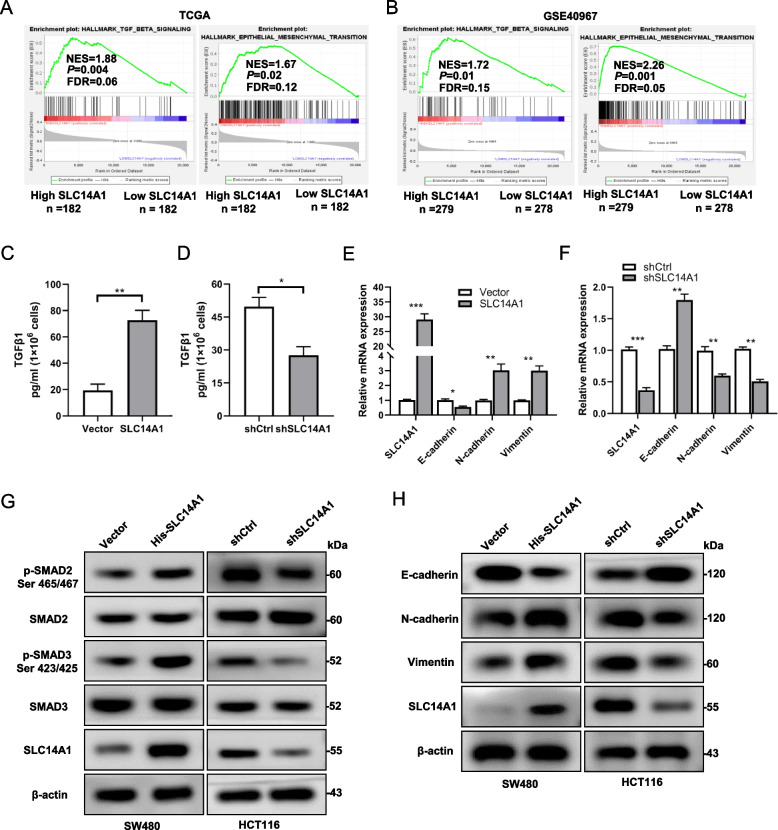
SLC14A1 Facilitates EMT and TGF-β Pathway Activation in CRC. **A**, **B** Gene Set Enrichment Analysis (GSEA) enrichment plots reveal significant activation of TGF-β and EMT pathways in CRC specimens with high SLC14A1 expression in TCGA and GSE40967 datasets. **C**,** D** Estimation of TGF-β1 in the culture medium of SW480 cell line overexpressing SLC14A1 or empty vector (**C**) and HCT116 cell line with SLC14A1 knockdown or control (**D**) using ELISA. Data are mean ± SEM, *, *P* < 0.05; **, *P* < 0.01. All data were obtained from at least three independent experiments. **E**,** F** Quantitative PCR (qPCR) analysis of the SLC14A1, E-cadherin, N-cadherin and Vimentin mRNA levels in SW480 (E) and HCT116 (F) cells with the indicated SLC14A1 modulation, three independent experiments, data were presented as the mean ± SEM, a 2-tailed, unpaired t test was used to determine statistical significance. *P < 0.05, **P < 0.01, ***P < 0.001. **G.** SLC14A1 overexpression enhanced, while its knockdown diminished, the activity of the TGF-β pathway in CRC cells. Western blot analysis was performed to assess the expression of relevant proteins in cells with overexpressed or knocked down SLC14A1, post-stimulation with TGF-β1 (1 ng/ml, 10 min). **H** Modulating SLC14A1 expression levels promoted or inhibited the EMT process in CRC cells. Western blot was utilized to analyze the expression of specific proteins in cells with overexpressed or knocked down SLC14A1, following TGF-β1 stimulation

We aimed to elucidate the molecular mechanisms underpinning the observed facilitation of CRCLM by SLC14A1. Stratification of patients from the TCGA and GSE40967 databases based on SLC14A1 expression levels facilitated a comprehensive Gene Set Enrichment Analysis (GSEA) on the transcriptional datasets of CRC patients. Remarkably, both databases unveiled a conspicuous enrichment of TGF-β and EMT pathway gene sets in patients with elevated SLC14A1 expression (Fig. 3A, B). This pointed towards a hypothetical model where the upregulation of SLC14A1 could be mechanistically linked to the activation of TGF-β signaling, which, in turn, orchestrates the EMT process pivotal for CRCLM. To validate our hypothesis, we initially analyzed the correlation between SLC14A1 and TGF-β signaling pathway target genes in the TCGA database. SLC14A1 showed a positive correlation with classical downstream targets of the TGF-β pathway such as TGFBI, CTGF, CDKN2B, and MMP2 (Additional file 2: Fig. S2A-D). Furthermore, using ELISA, we assessed the levels of soluble TGF-β in CRC cells with either overexpressed or knocked-down SLC14A1. Results indicated that overexpression of SLC14A1 increased soluble TGF-β levels in SW480 cells (Fig. 3C), whereas knockdown of SLC14A1 decreased these levels in HCT116 cells (Fig. 3D). Additionally, database analysis revealed a positive correlation between SLC14A1 and mesenchymal genes including N-Cadherin, Vimentin, ZEB1, and ZEB2 (Additional file 2: Fig. S2E-H). qPCR experiments confirmed that overexpression of SLC14A1 downregulated E-cadherin mRNA and upregulated N-cadherin and Vimentin mRNA levels in SW480 cells (Fig. 3E). In contrast, knockdown of SLC14A1 in HCT116 cells upregulated E-cadherin mRNA and downregulated N-cadherin and Vimentin mRNA levels (Fig. 3F). Western blot results were consistent with these findings; overexpression of SLC14A1 upregulated phosphorylated SMAD2 and SMAD3 levels, while knockdown suppressed these levels (Fig. 3G). Similarly, overexpression of SLC14A1 decreased E-cadherin protein levels and increased N-Cadherin and Vimentin protein levels, whereas knockdown of SLC14A1 had the opposite effects (Fig. 3H).

Collectively, these findings from both computational and experimental domains unequivocally assert the integral role of SLC14A1 upregulation in CRCLM, mechanistically mediated through the activation of the TGF-β pathway and the subsequent induction of the EMT process.

### SLC14A1 modulates transforming growth factor *beta* receptor 2 (TβRII) stability and ubiquitination in CRC

**Fig. 4:**
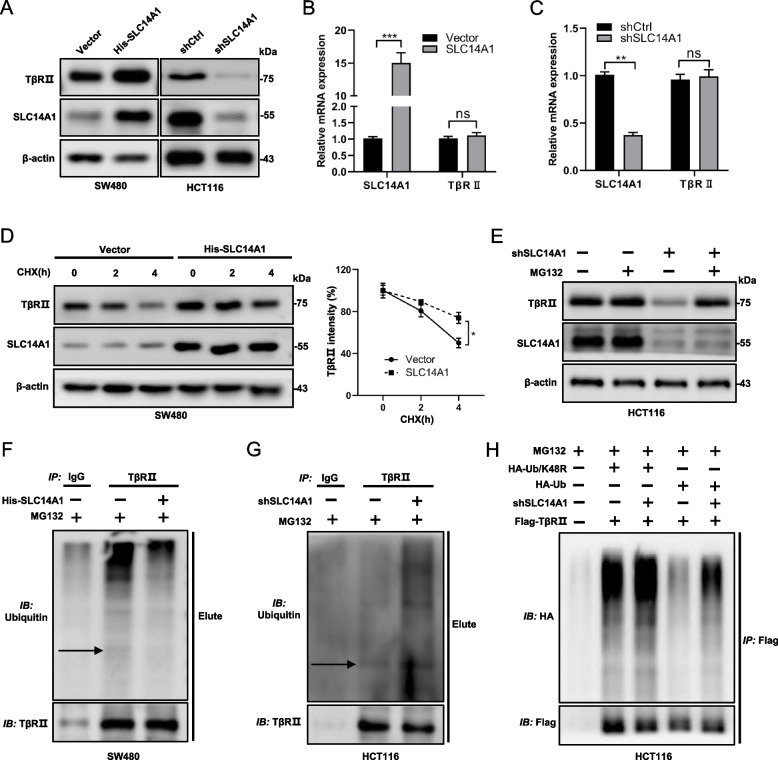
SLC14A1 Modulates TGF-β Pathway by Regulating TβRII Lys48-Linked Poly-Ubiquitination and Degradation. **A** SLC14A1 overexpression led to an increase in TβRII protein levels, while its knockdown resulted in decreased levels. Western blot analysis was used to examine TβRII protein in SW480 cells with SLC14A1 overexpression (left lane) and HCT116 cells with SLC14A1 knockdown (right lane). **B**, **C** SLC14A1 exerted no significant effect on TβRII mRNA expression. Quantitative PCR (qPCR) analysis of TβRII mRNA in SW480 or HCT116 cells with altered SLC14A1 expression. Results from three independent experiments are presented as mean ± SEM. A 2-tailed, unpaired t-test was used to determine statistical significance (***, *P* < 0.001, **, *P* < 0.01, ns, not significant). **D** SLC14A1 overexpression extended the half-life of TβRII. Western blot analysis in SW480 cells treated with cycloheximide (CHX) assessed the impact of SLC14A1 on TβRII stability. Protein half-life curves were derived from three independent experiments (right). **E** SLC14A1 influenced TβRII turnover via the proteasome pathway. Western blot analysis was performed to detect TβRII protein levels in HCT116 cells with SLC14A1 knockdown and treated with MG132 (20 μM) for 8 h before collection. **F**, **G** SLC14A1 overexpression inhibited, while its knockdown enhanced TβRII ubiquitination. Ubiquitination assays of TβRII in cell lysates from SW480 or HCT116 cells with specific SLC14A1 modulation. **H** SLC14A1 modulated TβRII ubiquitination specifically in Lys48-linked poly-ubiquitination. TβRII ubiquitination assays were conducted in HCT116 cells transfected with HA-Ub or HA-Ub/K48R and Flag- TβRII

While the manipulation of SLC14A1 exerted no tangible impact on the total protein levels of Smad2/3, a pronounced effect was observed on TβRII. Overexpression of SLC14A1 resulted in an increased protein level of TβRII, while its knockdown rendered an explicit reduction (Fig. 4A). Intriguingly, qPCR experiments revealed that neither overexpression nor knockdown of SLC14A1 in SW480 or HCT116 cells impacted the mRNA levels of TβRII (Fig. 4B, C), indicating a post-transcriptional regulatory mechanism.

Considering the documented evidence that TβRII protein is predominantly regulated through the ubiquitin–proteasome pathway [22, 23], offering a stringent control mechanism for the TGF-β pathway, we ventured to assess the influence of SLC14A1 on the stability of TβRII protein. Utilizing the protein synthesis inhibitor cycloheximide (CHX), we monitored TβRII protein levels at varying time points and observed that overexpression of SLC14A1 in the SW480 cell line extended the half-life of TβRII protein (Fig. 4D). Furthermore, the introduction of proteasome inhibitor MG132 neutralized the degradative effect induced by SLC14A1 knockdown on TβRII, supporting the hypothesis of ubiquitin-mediated degradation **(**Fig. 4E**)**. Immunoprecipitation of TβRII, followed by ubiquitin detection, unveiled that overexpressing SLC14A1 considerably reduced the ubiquitination levels of TβRII (Fig. 4F and Additional file 2: Fig. S3A), while its knockdown had an opposite, enhancing effect (Fig. 4G and Additional file 2: Fig. S3B). To confirm the type of ubiquitination mediated by SLC14A1, we employed the HCT116 cell model with SLC14A1 knockdown and co-transfected Flag-TβRII with either wild-type ubiquitin HA-Ub or the 48 lysine mutant ubiquitin HA-Ub/K48R. The subsequent immunoprecipitation and ubiquitination assays showed that SLC14A1 knockdown accelerated the ubiquitination of exogenous TβRII protein (wild-type) but no effect on the 48 lysine mutant ubiquitination (Fig. 4H and Additional file 2: Fig. S3C). This provided conclusive evidence that the ubiquitination of TβRII mediated by SLC14A1 is of the K48-linked ubiquitination.

### SLC14A1 inhibits ubiquitination of TβRII by competitively binding to Smurf1

**Fig. 5:**
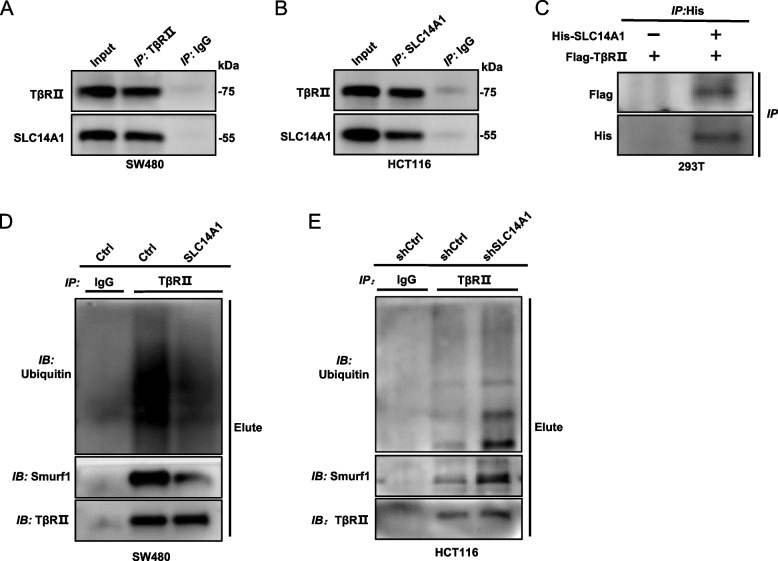
SLC14A1 Interacts with TβRII and Competitively Inhibits TβRII’s Association with E3 Ubiquitin Ligase Smurf1. **A**, **B** Interaction between SLC14A1 and TβRII was identified in CRC cells. Cell lysates were subjected to immunoprecipitation (IP) using anti-SLC14A1 antibodies or anti-TβRII, followed by Western blot analysis to detect TβRII or SLC14A1 in the co-immunoprecipitates. **C** Interaction between exogenous SLC14A1 and TβRII was confirmed in 293 T cells transfected with His-SLC14A1 and Flag-TβRII. Lysates were immunoprecipitated with anti-His antibodies, and the presence of Flag-tagged protein was detected. **D**,** E** SLC14A1 reduces the interaction of TβRII with Smurf1. In SW480 cells overexpressing SLC14A1 and HCT116 cells with SLC14A1 knockdown, anti-TβRII antibody-mediated IP was conducted. Western blot analysis was used to determine the presence of Smurf1 and other relevant proteins in the co-immunoprecipitates

Our subsequent immunoprecipitation experiments revealed an endogenous interaction between SLC14A1 and TβRII in the SW480 cell line (Fig. 5A), a finding further affirmed by reverse co-immunoprecipitation assays (Fig. 5B). Additionally, exogenous Co-IP experiments, involving the overexpression of His-SLC14A1 and Flag-TβRII in 293 T cell lines, validated this interactive relationship (Fig. 5C and Additional file 2: Fig. S3D).

Given that protein ubiquitination necessitates the combinative action of multiple proteins, including E3 ligases, we postulated that the SLC14A1-TβRII interaction could potentially modulate this complex process. Most previous studies implicates Smurf1 as the chief E3 ligases mediating TβRII-K48 linked ubiquitination [24, 25]. In this light, we endeavored to discern the influence of SLC14A1 manipulation on the interaction between TβRII and the Smurf1, and its subsequent effect on ubiquitination. Our findings unveiled that SLC14A1 overexpression attenuated the interaction between TβRII and Smurf1, concurrently inhibiting TβRII ubiquitination (Fig. 5D and Additional file 2: Fig. S3E). Conversely, knocking down SLC14A1 amplified the interaction and promoted ubiquitination (Fig. 5E and Additional file 2: Fig. S3F).

These outcomes unequivocally ascertain that SLC14A1, through its interactive dynamic with TβRII, impedes the binding of E3 ubiquitin ligase Smurf1 to TβRII. This intervention subsequently inhibits TβRII ubiquitination, augments its stability, and amplifies the activation of the TGF-β pathway.

### SLC14A1 is transcriptional activated by Snail

**Fig. 6:**
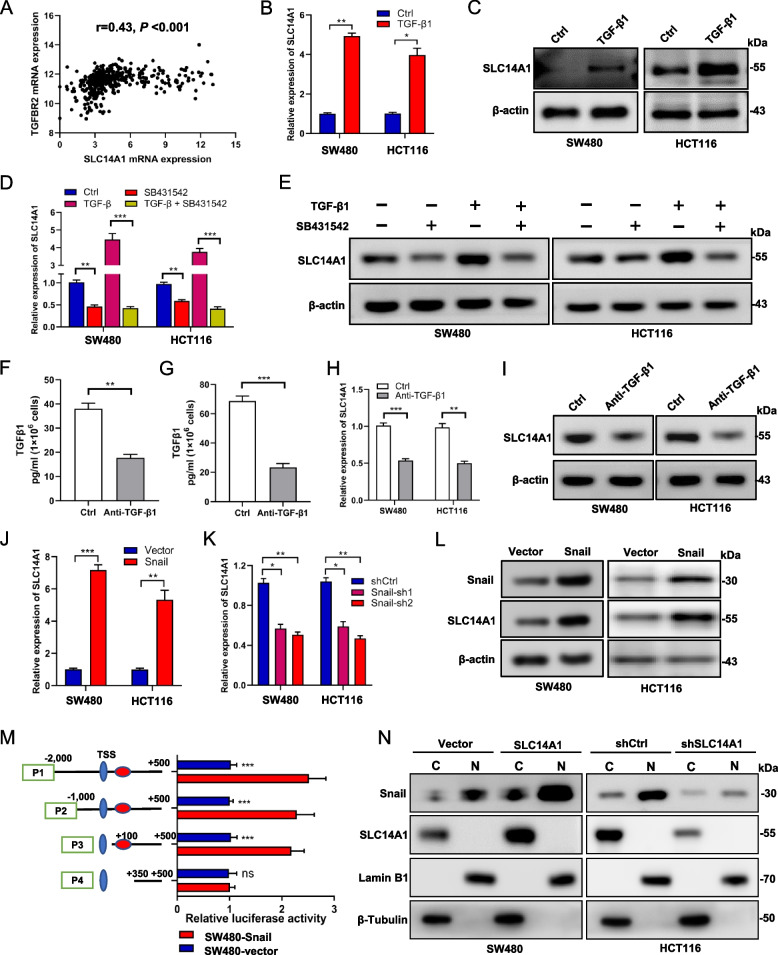
SLC14A1 Transcriptionally Activated by Snail. **A** Spearman correlation analysis displays the relationship between SLC14A1 and TβRII mRNA levels in the TCGA dataset. **B**,** C** Activation of the TGF-β pathway enhances SLC14A1 expression. **B** Quantitative PCR (qPCR) analysis demonstrates increased SLC14A1 mRNA in SW480 and HCT116 cells following treatment with TGF-β1 (1 ng/ml). Data are presented as mean ± SEM, and statistical significance was assessed using a 2-tailed, unpaired t-test (**P < 0.01, *P < 0.05). **C** Western blot analysis confirms elevated SLC14A1 protein levels post-TGF-β1 treatment (1 ng/ml). **D** qPCR analysis of SLC14A1 mRNA in SW480 and HCT116 cells in control, TGF-β1 (1 ng/ml), SB431542 (10 μM) and TGF-β1 + SB431542 groups. Data are presented as mean ± SEM, and statistical significance was assessed using a 2-tailed, unpaired t-test (***P < 0.001, **P < 0.01). **E** Western blot analysis of SLC14A1 protein levels in control, TGF-β1, SB431542 and TGF-β1 + SB431542 groups. **F, G.** Estimation of TGF-β1 in the culture medium of SW480 (**F**) and HCT116 (**G**) cell line treated with Anti-TGF-β1 antibody (20 μg/ml) or control using ELISA. Data are mean ± SEM, ***, *P* < 0.001; **, *P* < 0.01. All data were obtained from at least three independent experiments. **H** qPCR analysis of SLC14A1 mRNA in SW480 and HCT116 cells treated with Anti-TGF-β1 antibody or control. Data are mean ± SEM; statistical significance assessed via a 2-tailed, unpaired t-test (***P < 0.001, **P < 0.01). **I** Western blot analysis assesses SLC14A1 protein levels in SW480 and HCT116 treated with Anti-TGF-β1 antibody or control. **J**,** K** qPCR analysis of SLC14A1 mRNA in SW480 and HCT116 cells with Snail overexpression or knockdown. Data are mean ± SEM; statistical significance assessed via a 2-tailed, unpaired t-test (***P < 0.001, **P < 0.01, *P < 0.05). **L** Western blot analysis assesses SLC14A1 protein levels in SW480 and HCT116 cells after Snail overexpression. **M** The dual-luciferase reporter assay elucidates the interaction between specified regions of the SLC14A1 promoter and Snail. Data are mean ± SEM; statistical significance assessed via a 2-tailed, unpaired t-test (***P < 0.001, ns, not significant). **N** Western blot analysis was used to detect the protein expression of Snail and SLC14A1 in the cytoplasm and nucleus of SW480 cell line overexpressing SLC14A1 or empty vector (left lane) and HCT116 cell line with SLC14A1 knockdown or control (right lane). C, cytoplasm; N, nucleus

We initiated an investigation to understand the dysregulation of SLC14A1 expression levels within CRC tumor tissues. Although our preliminary analysis of TCGA data indicated no significant genomic alterations concerning SLC14A1, an intriguing observation was the significant positive correlation at the mRNA level between SLC14A1 and TβRII (Fig. 6A). Given that our earlier experiments established no significant impact of SLC14A1 manipulation on TβRII mRNA levels, this pointed towards the activation of the TGF-β pathway potentially enhancing SLC14A1 transcription.

To validate this hypothesis, we treated CRC cells with exogenous TGF-β1 and assessed the changes in SLC14A1 expression. Both qPCR and Western blot assays showed that TGF-β1 significantly increased the mRNA (Fig. 6B) and protein (Fig. 6C) levels of SLC14A1. Subsequently, we inhibited the TGF-β pathway using the TGF-β type I receptor kinase inhibitor SB431542 and further evaluated the expression of SLC14A1. The results from qPCR and Western blot indicated that the addition of SB431542 significantly attenuated the upregulation of SLC14A1 induced by TGF-β1 (Fig. 6D and E). Additionally, we explored the impact of the autocrine TGF-β pathway on SLC14A1 expression. ELISA results demonstrated that the addition of anti-TGF-β1 neutralizing antibodies to the SW480 or HCT116 cells culture medium significantly reduced the levels of TGF-β1 (Fig. 6F and G), and correspondingly, the mRNA (Fig. 6H) and protein (Fig. 6I) levels of SLC14A1 were decreased. These findings confirm that activation of the TGF-β pathway can promote the expression of SLC14A1.

Furthermore, we delved into the relationship between the core transcription factor of the TGF-β pathway, Snail, and SLC14A1. qPCR experiments revealed that the overexpression of Snail markedly increased SLC14A1 mRNA levels in CRC cells (Additional file 2: Fig. S4A andFig. 6J), while knocking down Snail led to a notable reduction (Additional file 2: Fig. S4B andFig. 6K). Western blotting experiments echoed these findings, indicating a significant upregulation of SLC14A1 protein levels with Snail overexpression (Fig. 6L) and a decrease upon Snail knockdown (Additional file 2: Fig. S4C, D). To pinpoint the specific binding site that facilitates the transcription of SLC14A1 by the transcription factor Snail, we analyzed the nucleotide sequence from 2000 bp upstream to 500 bp downstream of the SLC14A1 gene transcription start site. JASPAR database analysis indicated four potential Snail binding sites within this region (Additional file 2: Fig. S4E).

To elucidate the binding sites of Snail with the SLC14A1 gene, we cloned the full-length sequence of the SLC14A1 promoter [-2000 to + 500 bp relative to the transcription start site (TSS), P1] and truncated sequences (P2-P4) into the pGL3-Basic luciferase plasmid. Subsequent dual-luciferase assay results revealed that the luciferase activity of P1-P3 was increased in cells overexpressing Snail compared to control cells. In contrast, no significant difference in luciferase activity was observed for P4 between Snail-overexpressing and control cells (Fig. 6M). These results indicate that the regulatory site of Snail is located at + 150 to + 350 bp of the SLC14A1 promoter. Considering that Snail, as a transcription factor regulated by the activity of the TGF-β pathway, must localize to the nucleus to promote transcription of target genes, an important question arises: whether the changes in Snail expression induced by alterations in SLC14A1 expression primarily occur in the nucleus. To address this, we performed nuclear-cytoplasmic fractionation on CRC cells with overexpressed or knocked-down SLC14A1, and assessed the changes in Snail protein levels. The results showed that overexpression of SLC14A1 significantly increased the levels of Snail protein in the nucleus of SW480 cells (Fig. 6N, left lane), while knockdown of SLC14A1 markedly reduced the nuclear levels of Snail in HCT116 cells (Fig. 6N, right lane).

Collectively, these findings substantiate the existence of a positive feedback loop between SLC14A1 and the TGF-β pathway. During the progression of CRC, mutations in TGF-β pathway-related molecules trigger the activation of the pathway. This activation fosters the transcription of SLC14A1 by the downstream transcription factor Snail. Elevated SLC14A1 levels, in turn, inhibit TβRII degradation via the ubiquitin–proteasome pathway, maintaining the TGF-β pathway in an activated state. This sustained activation facilitates the EMT process, culminating in CRCLM.

### Clinical correlation between SLC14A1, Phospho-Smad2, and Snail in human CRC tissues

**Fig. 7:**
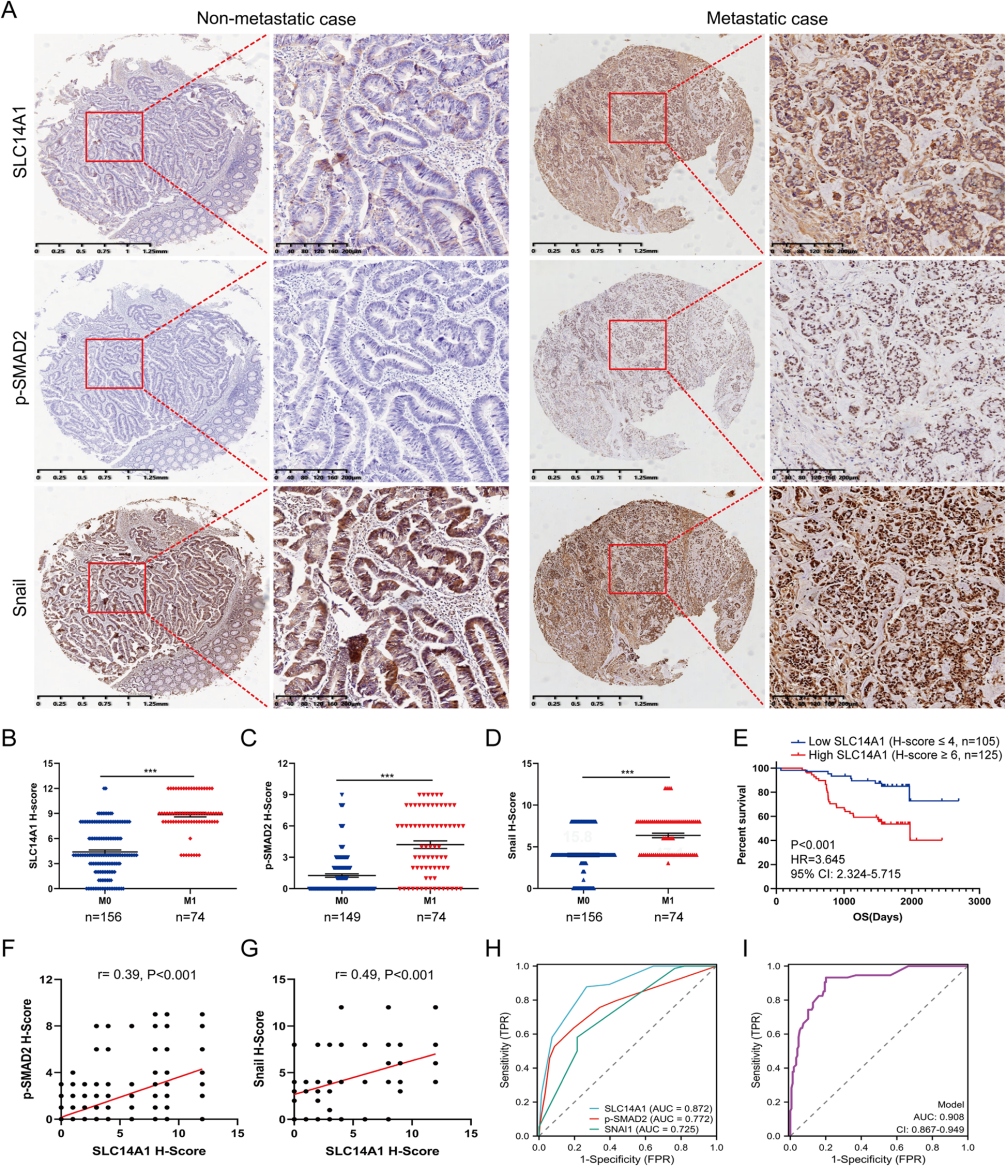
Elevated SLC14A1 Protein Levels Correlate with CRC Metachronous Liver Metastasis and Serve as a Prognostic Marker. **A-D** Immunohistochemical (IHC) analysis of SLC14A1, phosphorylated Smad2 (p-SMAD2), and Snail in CRC specimens from 230 patients. **A** Representative IHC images illustrate staining differences in cases with metachronous liver metastasis compared to those without. Scale bars: 1.25 mm. Right panels show magnified views of the areas highlighted by dashed lines on the left. Scale bars: 200 μm. **B-D** Quantitative evaluation of the H-score for SLC14A1, p-SMAD2, and Snail in CRC specimens. Data are presented as mean ± SEM, with statistical significance assessed using the Mann–Whitney test (*, *P* < 0.05, **, *P* < 0.01). **E** Kaplan–Meier (KM) survival curves depict overall survival (OS) stratified by SLC14A1 H-score in CRC patients, analyzed using the log-rank test. **F**,** G** Spearman correlation analysis evaluates the association between the H-scores of SLC14A1 and those of p-SMAD2 or Snail in CRC specimens. **H** Receiver operating characteristic (ROC) curves for the H-scores of SLC14A1, p-SMAD2, and Snail as predictive tools for metachronous liver metastasis in CRC patients. **I** A combined ROC curve for the H-scores of SLC14A1, p-SMAD2, and Snail enhances predictive accuracy for metachronous liver metastasis in CRC patients

To validate the theoretical constructs elucidated in our study on a clinical level, IHC detection of SLC14A1, phosphorylated Smad2 (p-SMAD2), and Snail was conducted on postoperative tumor tissues from 230 CRC patients (Fig. 7A). The findings revealed that patients with metachronous liver metastasis exhibited significantly higher IHC scores (H-Score) for SLC14A1 compared to those without metachronous liver metastasis (Fig. 7B). Similarly, the H-Scores for p-SMAD2 (Fig. 7C) and Snail (Fig. 7D) were also notably elevated in the metachronous liver metastasis patient group. Next, patients were classified into high and low SLC14A1 expression groups based on H-Scores, using thresholds of greater than or equal to 6 and less than or equal to 4, respectively. Prognostic differences between these two groups were then analyzed. The Kaplan–Meier survival curves demonstrated that patients with high SLC14A1 expression manifested a significantly poorer overall survival compared to the low SLC14A1 expression group (Fig. 7E). A correlation analysis revealed a significant positive association between the H-Scores of SLC14A1 and those of p-SMAD2 and Snail (Fig. 7F and G).

Furthermore, we investigate the association of SLC14A1 with clinicopathological features of CRC, such as gender and age of patients, differentiation, TNM stage, adjuvant chemotherapy, metachronous liver metastasis, as well as H-Scores of p-SMAD2 and Snail. Our data showed that a high level of SLC14A1 was correlated with presence of metachronous liver metastasis, and high H-Scores of p-SMAD2 and Snail (Table 1). To assess the efficacy of using the H-Scores of these three molecules to predict metachronous liver metastasis in CRC patients in clinical practice, corresponding receiver operating characteristic (ROC) curves were plotted. The results demonstrated that the area under the curve (AUC) for SLC14A1 was 0.872, which was higher than that for p-SMAD2 and Snail at 0.772 and 0.725, respectively (Fig. 7H). Additionally, the combined ROC curve showed that the efficacy of predicting metachronous liver metastasis using a composite of these three indicators reached 0.908 (Fig. 7I).

**Table 1:** Relationship of SLC14A1 expression level with CRC clinicopathological features

	Low SLC14A1 (H-score≤4)	High SLC14A1 (H-score≥6)	X^2^	*P* Value
Variable	*n*=105	*n*=125		
Age(year)				
≥ 60	43	62	1.72	0.19
<60	62	63		
Gender				
Male	60	68	0.17	0.68
Female	45	57		
Differentiation				
High	102	116	2.18	0.14
Moderate or Low	3	9		
TNM stage				
I	3	5	0.24	0.89
II	16	18		
III	86	102		
Adjuvant chemotherapy				
FOLFOX	43	54	0.89	0.64
CAPEOX	34	50		
FOLFIRI	3	7		
Metachronous Liver Metastases				
Absent	87	69	20.00	<0.0001
Present	18	56		
p-SMAD2 H-score				
Low (H-score≤4)	90	88	15.67	<0.0001
High (H-score≥6)	8	37		
Snail H-score				
Low (H-score≤4)	88	70	20.52	<0.0001
High (H-score≥6)	17	55		

These clinical findings align with our prior theoretical and experimental results, reinforcing the critical role of SLC14A1, in tandem with the activation of the TGF-β pathway and EMT process, in promoting metachronous liver metastasis in CRC patients. The elevated expression of SLC14A1, phospho-Smad2, and Snail not only corroborates their synergistic interaction but also underscores their potential as significant biomarkers and therapeutic targets for managing CRC progression and metastasis.

## Discussion

In the realm of clinical practice, the management of metachronous liver metastasis in CRC presents a formidable challenge. On one hand, metachronous liver metastasis exhibits limited responsiveness to systemic chemotherapy and current targeted therapies, such as cetuximab and bevacizumab [26, 27]. On the other hand, there is presently a dearth of effective molecular markers to predict the occurrence of metachronous liver metastasis in postoperative patients [28]. Therefore, the further elucidation of the molecular mechanisms underlying metachronous liver metastasis in CRC holds significant importance in identifying novel therapeutic targets and screening for predictive biomarkers of liver metastasis.

In our pursuit of this inquiry, we conducted a comprehensive DEGs analysis on two independent databases, which led to the identification of two genes, SLC14A1 and GSR, associated with metachronous liver metastasis in CRC. The principal biological function of GSR (glutathione-disulfide reductase) lies in catalyzing the reduction of oxidized glutathione (GSSG) to reduced glutathione (GSH), thereby upholding the intracellular antioxidant defense system [29]. Several studies have indicated that heightened expression of GSR is linked to the invasiveness and poor prognosis of certain cancer types, likely attributable to the bolstered antioxidant capabilities of tumor cells with elevated GSR levels, enabling them to better adapt to and counter adverse extracellular environments [30, 31]. In the context of liver metastasis, GSR may foster the survival and proliferation of tumor cells in the liver by aiding their resistance to the distinct oxidative stress encountered in liver tissue [32].

Our investigation delved deeply into the relationship between the SLC14A1 gene and metachronous liver metastasis in CRC. Through in vitro cell experiments, we observed that SLC14A1 enhances the invasion and migration capabilities of CRC cells. Crucially, this finding was substantiated at the in vivo experimental level through cecal orthotopic injection and splenic injection of CRC cells in a mouse model of liver metastasis. Moreover, both the analysis of CRC patients in the database and the examination of postoperative specimens from our collected cohort confirmed the close association of upregulated SLC14A1 with metachronous liver metastasis and poor prognosis in patients. Regarding the relationship between SLC14A1 and other tumor types, several studies have indicated its potential distinctive function in specific cancer forms. For instance, Ye et al. reported that SLC14A1 is markedly downregulated in prostate cancer, and patients with high SLC14A1 expression have a longer biochemical recurrence-free period, indicating that SLC14A1 may exert an anti-cancer effect in the prostate and is mediated through specific miRNAs and B lymphocyte infiltration [33]. In bladder cancer, the chromosomal region containing the SLC14A1 gene, at 18q12.3, has been identified as a susceptibility locus for bladder cancer, and variations in the SLC14A1 gene may offer a novel etiological perspective on bladder cancer occurrence [34, 35]. Additionally, Professor Ma and his colleagues discovered that in bladder cancer, patients with a high proportion of SLC14A1^**+**^ cancer-associated fibroblast (CAF) exhibited a poorer response to neoadjuvant chemotherapy and immunotherapy. Mechanistic studies revealed that the SLC14A1^**+**^ subtype of CAF in bladder cancer originates from interferon-stimulated differentiation and can secrete WNT5A, thereby activating the Wnt pathway in bladder cancer cells in a paracrine manner and enhancing tumor cell stemness [18]. It is noteworthy that the upregulation of SLC14A1 in this subtype of CAF is an outcome of its concomitant upregulation induced by interferon stimulation, and SLC14A1 itself does not regulate the secretion of WNT5A. Nonetheless, SLC14A1^**+**^ CAF can still serve as a target for improving the treatment responsiveness of bladder cancer patients. In present study, we conducted an analysis of single-cell sequencing data from CRC samples in the GSE245552 database to explore whether SLC14A1^**+**^ CAF are also present in CRC. The results of our analysis revealed that the CAFs could be classified into 5 distinct subtypes through dimensionality reduction clustering. Interestingly, none of these 5 subtypes exhibited characteristic expression of SLC14A1 (Additional file 2: Fig. S5 and Additional file 3: Table S2). This finding suggests that the phenomenon observed in bladder cancer, where SLC14A1^+^ CAF enhance the stemness of bladder cancer cells, is not present in CRC. Our results indicate a unique molecular landscape in CRC compared to bladder cancer, underscoring the potential differences in tumor microenvironment and cellular interactions between these two types of malignancies.

Our GSEA analyses suggest that SLC14A1 is intricately woven with the threads of TGF-β signaling and EMT, two pivotal elements notoriously implicated in cancer metastasis. SLC14A1 emerged not just as a bystander but as a potent catalyst in the activation of TGF-β signaling. Our mechanistic explorations unveiled its interaction with TβRII, offering a competitive edge that effectively inhibited Smurf1-mediated ubiquitination and subsequent degradation [25, 36, 37]. SLC14A1’s mechanism of action, particularly its role in impairing TβRII ubiquitination, extends our understanding of its functional repertoire. Previous reports, albeit limited, have not comprehensively explored this aspect. In existing literature, SLC14A1’s functionality has been primarily associated with urea transport and bladder cancer susceptibility, with minimal insights into its regulatory role in cellular signaling pathways [38–40].

The emergence of a positive feedback loop between SLC14A1 and the TGF-β pathway, as elucidated in our findings, is another significant milestone. The interaction of Snail, a downstream effector of TGF-β signaling [41, 42], with the SLC14A1 gene at a proximal downstream region was illustrative of a self-perpetuating mechanism driving sustained pathway activation. The positive correlation between SLC14A1 and phosphorylated Smad2, and Snail, validated in clinical specimens, underscores the clinical relevance of our findings.

The unveiling of SLC14A1's role in CRC metastasis opens up an avenue for potential therapeutic innovations. Targeting its interaction dynamics with TβRII could dampen TGF-β signaling, mitigate EMT progression, and potentially attenuate metastatic processes. Furthermore, the variability in SLC14A1 expression among individuals beckons towards a personalized therapeutic approach, where interventions are meticulously tailored to align with patients’ unique molecular profiles. Yet, as with any scientific exploration, this study is not devoid of limitations. The intricate interactions within the TGF-β signaling cascade and its interactions with other cellular pathways necessitate a more in-depth exploration. Furthermore, translating these findings from the bench to bedside mandates comprehensive in vivo functional validations and clinical trials.

## Conclusions

The present study elucidates that upregulation of SLC14A1 in the primary lesions of CRC activates the TGF-β pathway. This activation leads to a positive feedback loop with Snail, enhancing the metastatic capabilities of CRC cells. More importantly, the upregulation of SLC14A1 is closely associated with metachronous liver metastasis and poor prognosis in CRC patients, indicating its potential as a predictive marker for postoperative metachronous liver metastasis in these patients. These findings emphasize the prospective use of SLC14A1 as a predictive marker and therapeutic target to personalize treatment for CRC patients, facilitating improved clinical decision-making.

### Supplementary Information

Additional file 1:Table S1. The sequence of primers.

Additional file 2:SLC14A1 overexpression facilitated liver metastasis in a splenic injection liver metastasis model. (A-C) Schematic representation of CRC metastatic growth in the liver model induced by intrasplenic injection of the SW480 cells. BALB/C mice (*n* = 8) were injected splenically with either SW480/vector or SW480/SLC14A1 cells. After 14 days, mice were sacrificed, and the liver metastatic nodules were counted and statistically analyzed using a t-test (*, *P* < 0.05). Figure S2. SLC14A1 positively regulates the TGF-β pathway and EMT. (A-D) Spearman correlation of SLC14A1 and target genes of TGF-β pathway, such as TGFBI, CTGF, CDKN2B, and MMP2, at the mRNA level in the TCGA dataset. (E–H) Spearman correlation analysis of SLC14A1 and EMT-related genes, such as N-cadherin, Vimentin, ZEB1, and ZEB2, at the mRNA level in the TCGA dataset. Figure S3. The whole lysate images of cells from Fig. 4F-H and Fig. 5C-E. (A) The whole lysate of SW480 cell in Fig. 4F. (B) The whole lysate of HCT116 cell in Fig. 4G. (C) The whole lysate of HCT116 cells in Fig. 4H. (D) The whole lysate of 293 T cells in Fig. 5C. (E) The whole lysate of SW480 cell in Fig. 5D. (F) The whole lysate of HCT116 cell in Fig. 5E. Figure S4. Snail regulates the transcription of SLC14A1**.** (A, B) qPCR analysis of Snail mRNA in SW480 and HCT116 cells with Snail overexpression or knockdown. Data are mean ± SEM; statistical significance assessed via a 2-tailed, unpaired t-test (***P < 0.001, **P < 0.01, *P < 0.05). (C, D) Western blot analysis assesses SLC14A1 protein levels in SW480 and HCT116 cells after Snail knockdown. (E) Identification of the Snail binding motif predicted by the JASPAR database. Figure S5. Cancer-associated Fibroblast (CAF) typing analysis did not enrich SLC14A1^+^ CAF. (A) UMAP plot of cells in CRC samples from GSE245552 dataset. (B) Cell distribution in CRC samples depicted via UMAP. (C) UMAP plot of CAF in CRC samples. (D) Heatmap of top differentially expressed genes (DEGs) between each CAF subclusters.

Additional file 3:Table S2. DEGs of 5 types of CAF in GSE245552 database.

## Data Availability

The datasets used and/or analyzed during the current study are available from the corresponding author upon reasonable request. The public datasets analyzed in this study were obtained from the following sources: TCGA (Colorectal Adenocarcinoma, PanCancer Atlas), GSE40967 and GSE245552.

## Abbreviations

AUC: Area under curve
CAF: Cancer-associated fibroblast
CHX: Cyclohexamide
CI: Confidence interval
CoIP: Co-immunoprecipitation
CRC: Colorectal cancer
CRCLM: Colorectal cancer liver metastasis
DEGs: Differentially expressed genes
ELISA: Enzyme-linked immunosorbent assay
EMT: Epithelial-Mesenchymal Transition
GEO: Gene Expression Omnibus
GSEA: Gene set enrichment analysis
GSR: Glutathione-disulfide reductase
H-score: Histochemistry score
IHC: Immunohistochemical
KM: Kaplan–Meier
OS: Overall survival
qPCR: Quantitative real-time-PCR
RFS: Recurrence-free survival
ROC: Receiver operating characteristic
siRNA: Small interfering RNA
SLC14A1: Solute Carrier Family 14 Member 1
TCGA: The cancer genome atlas
TGF-β: Transforming growth factor beta
TβRII: Transforming growth factor beta receptor 2
TSS: Transcription start site
