# Supplementary material for: SLC14A1 and TGF-β signaling: a feedback loop driving EMT and colorectal cancer metachronous liver metastasis
Source: J Exp Clin Cancer Res. 2024 Jul 27;43:208. doi: 10.1186/s13046-024-03114-8 (PMC11282742; doi:10.1186/s13046-024-03114-8)
Supplement: Supplementary file 1 — Additional file 1:Table S1. The sequence of primers. [file 13046_2024_3114_MOESM1_ESM.docx]

| Gene | Primers |
| --- | --- |
| SLC14A1 | Forward (5’-3’): TTGCTCAGCCAGGACAGGTC  Reverse (5’-3’): GGAGTAACAGCCACCAGAAATAGTC |
| TβRⅡ | Forward (5’-3’): GCCAACAACATCAACCACAACAC  Reverse (5’-3’): GCCACTGTCTCAAACTGCTCTG |
| SNAI1 | Forward (5’-3’): CTCGCTGCCAATGCTCATCTG  Reverse (5’-3’): AGCCTTTCCCACTGTCCTCATC |
| ACTB | Forward (5’-3’): CACCATTGGCAATGAGCGGTTC  Reverse (5’-3’): AGGTCTTTGCGGATGTCCACGT |
| Vimentin | Forward (5’-3’): TGCGCCAGCAGTATGAAA  Reverse (5’-3’): GCCTCAGAGAGGTCAGCAAA |
| E-cadherin | Forward (5’-3’): CGAGAGCTACACGTTCACGG  Reverse (5’-3’): GGGTGTCGAGGGAAAAATAGG |
| N-cadherin | Forward (5’-3’): CCTCCAACGGGCATCTTCAT  Reverse (5’-3’): TGTCCACTGCATGTGCTCTC |

**Table S1.** Primer sequences for real-time RT-PCRs used in this study
