# Supplementary material for: SLC14A1 and TGF-β signaling: a feedback loop driving EMT and colorectal cancer metachronous liver metastasis
Source: J Exp Clin Cancer Res. 2024 Jul 27;43:208. doi: 10.1186/s13046-024-03114-8 (PMC11282742; doi:10.1186/s13046-024-03114-8)

Supplementary figure 1

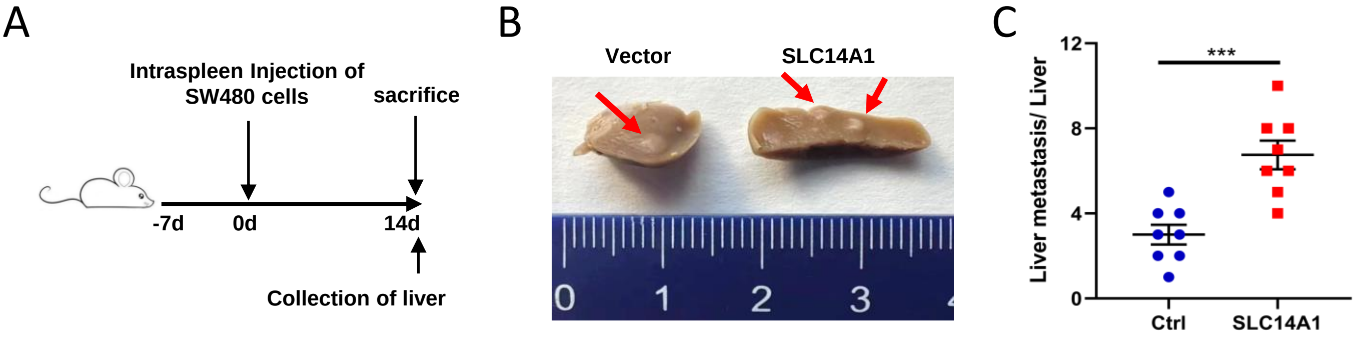

Supplementary figure 2

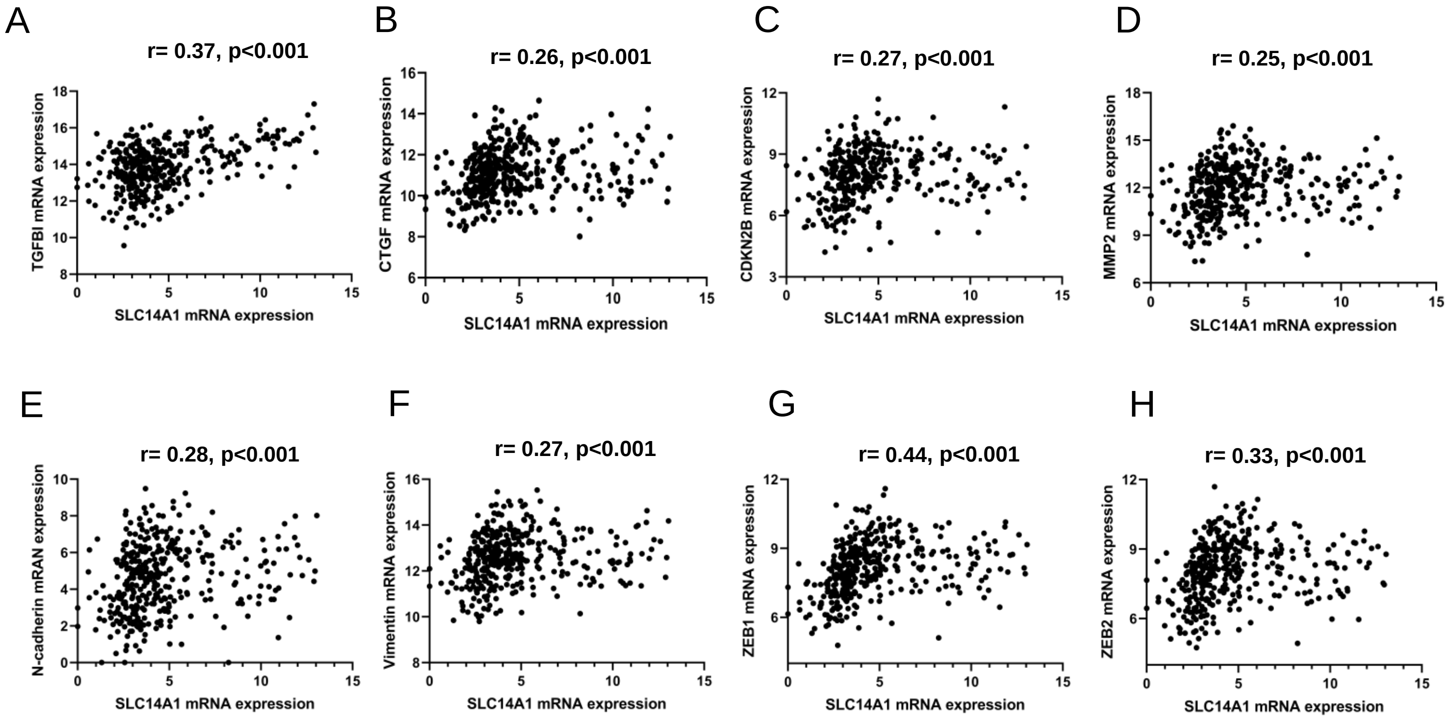

Supplementary figure 3

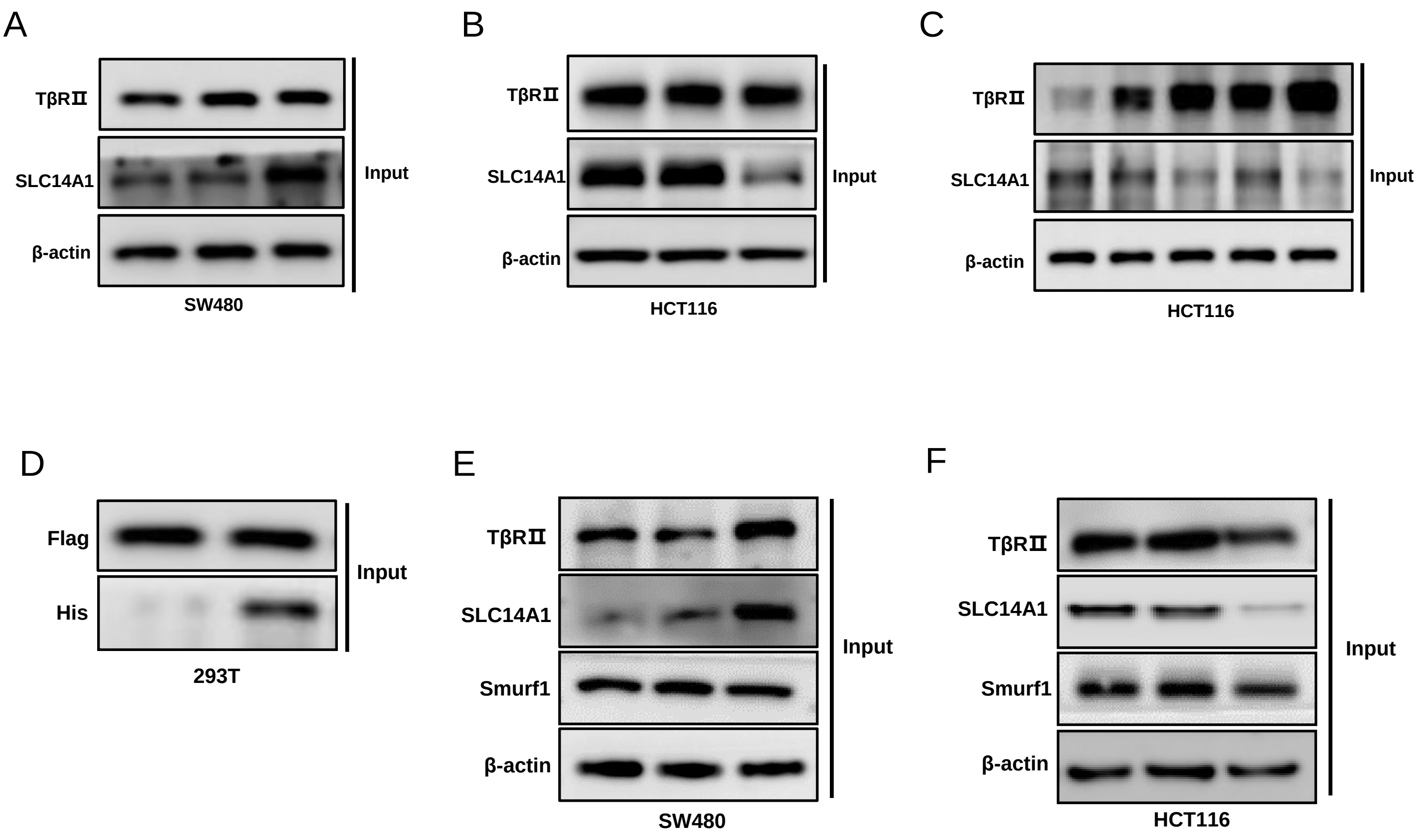

Supplementary figure 4

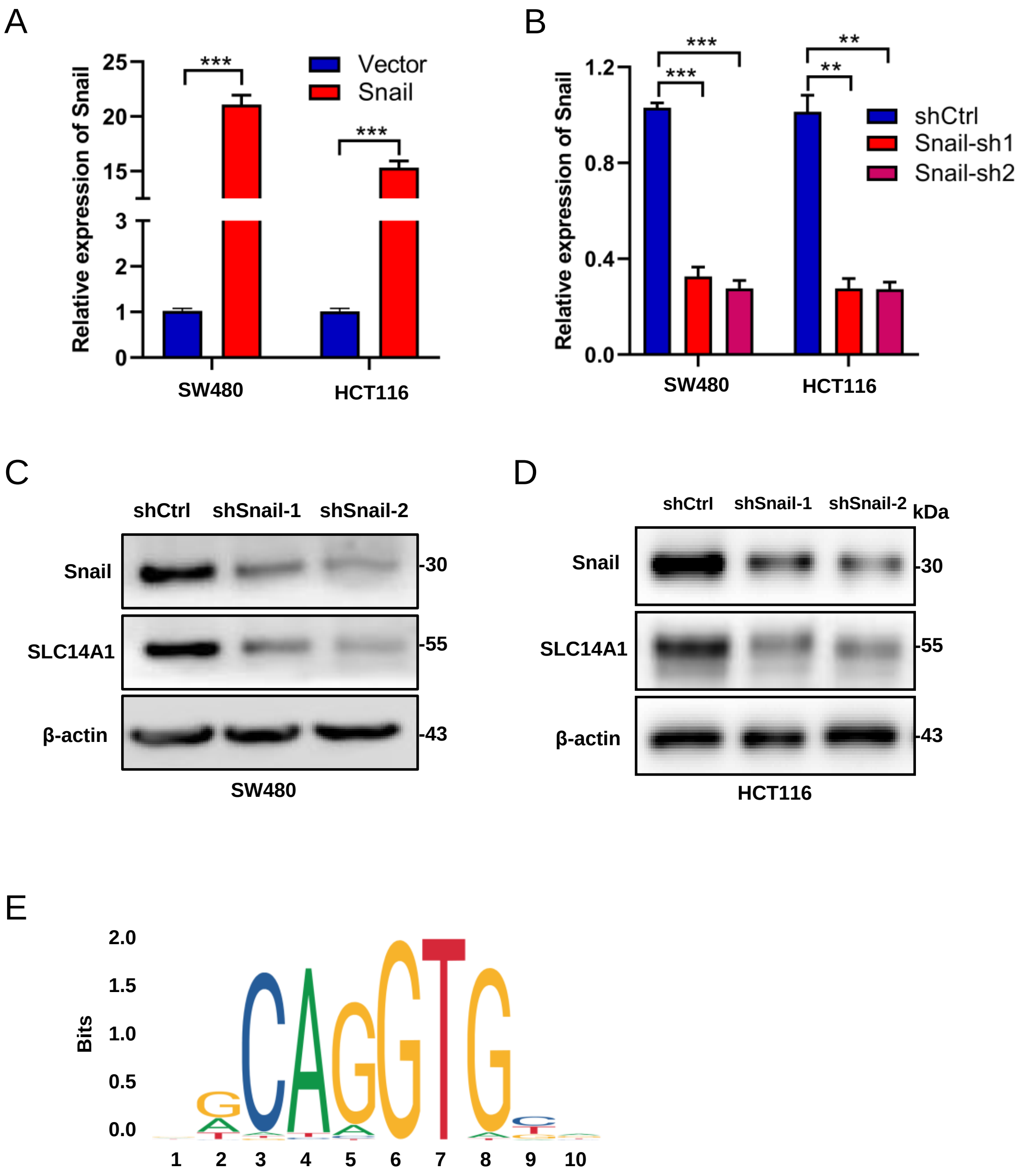

Supplementary figure 5

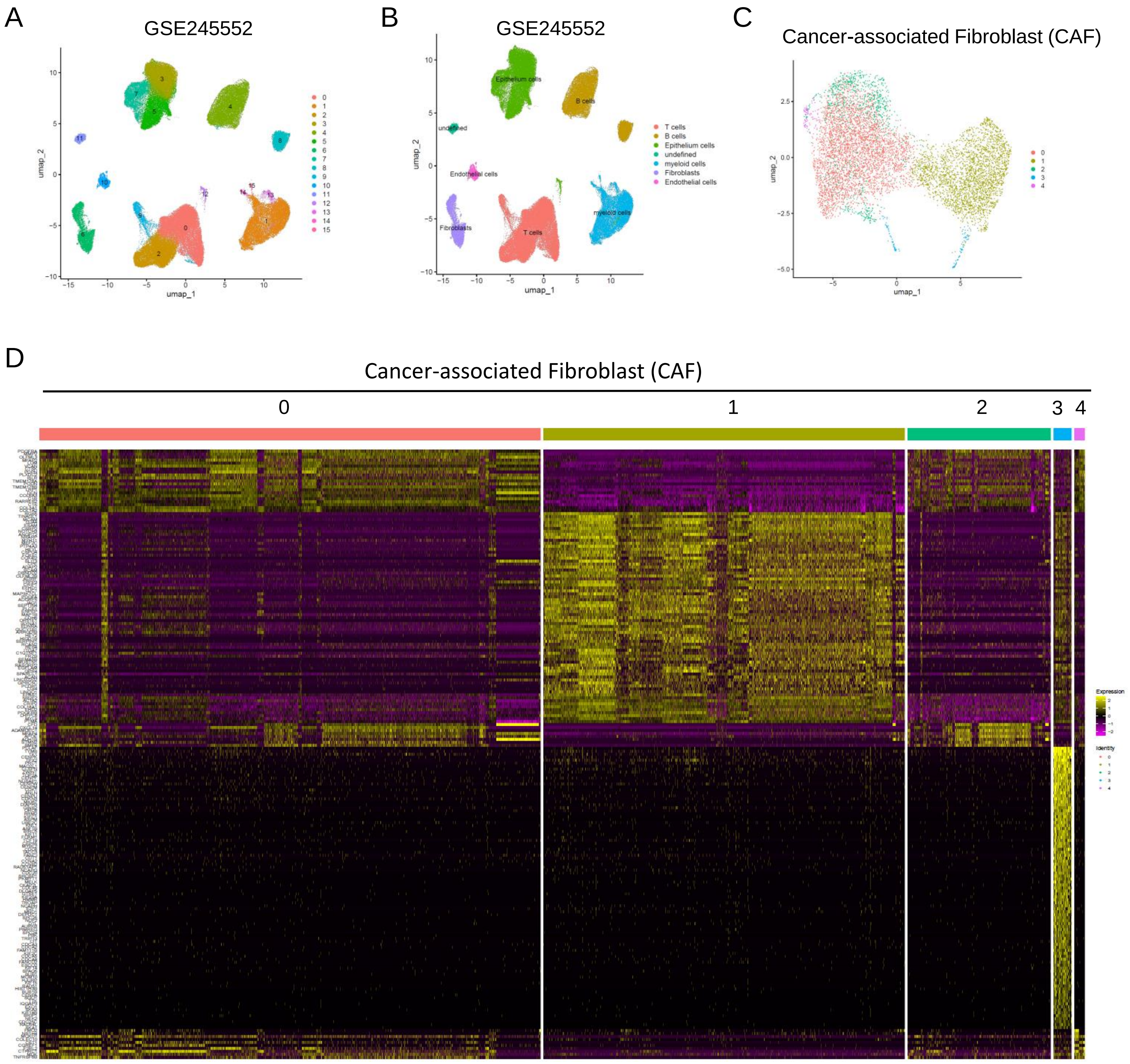

Supplement: Supplementary file 2 — Additional file 2:SLC14A1 overexpression facilitated liver metastasis in a splenic injection liver metastasis model. (A-C) Schematic representation of CRC metastatic growth in the liver model induced by intrasplenic injection of the SW480 cells. BALB/C mice (n = 8) were injected splenically with either SW480/vector or SW480/SLC14A1 cells. After 14 days, mice were sacrificed, and the liver metastatic nodules were counted and statistically analyzed using a t-test (*, P < 0.05). Figure S2. SLC14A1 positively regulates the TGF-β pathway and EMT. (A-D) Spearman correlation of SLC14A1 and target genes of TGF-β pathway, such as TGFBI, CTGF, CDKN2B, and MMP2, at the mRNA level in the TCGA dataset. (E–H) Spearman correlation analysis of SLC14A1 and EMT-related genes, such as N-cadherin, Vimentin, ZEB1, and ZEB2, at the mRNA level in the TCGA dataset. Figure S3. The whole lysate images of cells from Fig. 4F-H and Fig. 5C-E. (A) The whole lysate of SW480 cell in Fig. 4F. (B) The whole lysate of HCT116 cell in Fig. 4G. (C) The whole lysate of HCT116 cells in Fig. 4H. (D) The whole lysate of 293 T cells in Fig. 5C. (E) The whole lysate of SW480 cell in Fig. 5D. (F) The whole lysate of HCT116 cell in Fig. 5E. Figure S4. Snail regulates the transcription of SLC14A1. (A, B) qPCR analysis of Snail mRNA in SW480 and HCT116 cells with Snail overexpression or knockdown. Data are mean ± SEM; statistical significance assessed via a 2-tailed, unpaired t-test (***P < 0.001, **P < 0.01, *P < 0.05). (C, D) Western blot analysis assesses SLC14A1 protein levels in SW480 and HCT116 cells after Snail knockdown. (E) Identification of the Snail binding motif predicted by the JASPAR database. Figure S5. Cancer-associated Fibroblast (CAF) typing analysis did not enrich SLC14A1+ CAF. (A) UMAP plot of cells in CRC samples from GSE245552 dataset. (B) Cell distribution in CRC samples depicted via UMAP. (C) UMAP plot of CAF in CRC samples. (D) Heatmap of top differentially expressed genes (DEGs) between [file 13046_2024_3114_MOESM2_ESM.pdf]
